# Alterations of Fungal Microbiota in Patients With Cholecystectomy

**DOI:** 10.3389/fmicb.2022.831947

**Published:** 2022-05-11

**Authors:** Jun Xu, Xinhua Ren, Yun Liu, Yuanyuan Zhang, Yiwen Zhang, Guodong Chen, Qing Huang, Qing Liu, Jianhua Zhou, Yulan Liu

**Affiliations:** ^1^Department of Gastroenterology, Peking University People's Hospital, Beijing, China; ^2^Clinical Center of Immune-Mediated Digestive Diseases, Peking University People's Hospital, Beijing, China; ^3^Center of Liver Diseases, Beijing Ditan Hospital, Capital Medical University, Beijing, China; ^4^Institute of Clinical Molecular Biology and Central Laboratory, Peking University People's Hospital, Beijing, China

**Keywords:** fungal microbiota, cholecystectomy, carcinogenesis, *Candida glabrata* (*C. glabrata*), *Candida*

## Abstract

Increasing evidence suggests a high risk of gastrointestinal postoperative comorbidities (such as colorectal cancer) in patients with postcholecystectomy (PC). Although previous studies implicated the role of fungi in colon carcinogenesis, few reports focused on the fungal profile in patients with PC. We enrolled 104 subjects, including 52 patients with PC and 52 non-PC controls (CON), for fecal collection to detect the fungal composition by an internal transcribed spacer (ITS) 1 rDNA sequencing. Data showed that *Candida* (C.) *glabrata* and *Aspergillus* (A.) *Unassigned* were enriched, and *Candida albicans* was depleted in patients with PC. In addition, postoperative duration was the main factor to affect the fungal composition. Machine learning identified that *C. glabrata, A. Unassigned*, and *C. albicans* were three biomarkers to discriminate patients with PC from CON subjects. To investigate the fungal role in colon carcinogenesis, the subjects of the PC group were divided into two subgroups, namely, patients with PC without (non-CA) and with precancerous lesions or colorectal cancer (preCA_CRC), by histopathological studies. *C. glabrata* was found to be gradually accumulated in different statuses of patients with PC. In conclusion, we found fungal dysbiosis in patients with cholecystectomy, and the postoperative duration was a potent factor to influence the fungal composition. The accumulation of *C. glabrata* might be connected with carcinogenesis after cholecystectomy.

## Introduction

Cholecystectomy is the most popular operation for patients with biliary suffers, such as acute and chronic cholecystitis and gallstones. Notably, increasing evidence suggests a high risk of gastrointestinal postoperative comorbidities in patients with postcholecystectomy (PC) as gastrointestinal comorbidities, especially in colorectal cancer (CRC) within the first 5 years (Schernhammer et al., 2003; Shao and Yang, 2005; Chen et al., 2014; Li and Apte, 2015; Simpson, 2019). Changed luminal bile acid profile and gut microbiome tend to be two culprits in gastrointestinal inflammation and carcinogenesis (Jia et al., 2018). Previous data showed an aging-associated fecal commensal bacteria in patients with PC and then identified several bile acid metabolism-related bacteria as contributors to colorectal cancer incidence *via* elevation of secondary bile acids, such as *Bacteroides* (Wang T. et al., 2018). Our published data also showed a dramatic alteration in bacterial composition and patients with PC. We noticed that there was an abundant increase in *Bacteroides, Fusobacterium*, and *Megamonas* and a decrease in *Faecalibacterium, Bifidobacterium*, and *Prevotella* in patients with PC (Ren et al., 2020). Interestingly, these changes were accordant to the bacterial characterization of patients with CRC (Bullman et al., 2017; York, 2017; Yu et al., 2017; Yachida et al., 2019). These reports primarily showed an essential role of bacterial microbiota in carcinogenesis of long-term clinical results in patients with PC. Apart from bacteria, however, fungal pathogens were also reported to trigger the stage of colorectal carcinogenesis (Conche and Greten, 2018). Malik et al. (2018) and Wang T. et al. (2018) highlighted the role of adaptor protein CARD9 in defending commensal fungi. *Card9*-deficient mice are susceptible to various fungal infections, such as *C. albicans, Aspergillus fumigatus*, and *Cryptococcus neoformans*, subsequently undergoing an increased tumor load in *Card*9^−/−^ mice. Although the carcinogenic role of fungal contents is clear, few reports focused on the fungal role in the pathophysiological process of patients with PC. Thus, we focused on the fungal features in patients with PC and the carcinogenic process of patients after cholecystectomy.

## Methods

### Ethical Statement

This study was approved by the Institutional Medical Ethics Review Board of Peking University People's Hospital (Document No. 2018PHB035-01). All subjects provided written informed consent.

### Study Subjects

We enrolled 104 subjects for this study, including 52 patients with PC and 52 non-PC controls (CON) in Peking University People's Hospital from January 2018 to October 2018. Patients whose gallbladder had been removed for more than 6 months and <25 years were enrolled in the PC group. Based on the histopathology, patients with PC accompanied by precancerous lesions or colorectal cancer were assigned to a subgroup of preCA_CRC (*n* = 9). The others without precancerous lesions or colorectal cancer were set to the non-CA (*n* = 43) subgroup. The demographic data and primary clinical data of all subjects were recorded. All enrolled subjects were asked to avoid yogurt, mushrooms, agaric, and other fungal food within 1 week and avoid using probiotics and antibiotics at least 2 weeks before sampling. Every patient with PC received a colonoscopy. Notably, 28 patients with PC (53.8%) had undergone colonoscopy in our hospital or the other hospitals of the same level within 6 months, and the sampling was after the colonoscopy and intestinal lavage at least 1 month. A total of 24 patients with PC (46.2%) had no colonoscopy within 6 months, and the stool sampling was collected 1 day before the colonoscopy and intestinal lavage to avoid the effect of intestinal lavage on the gut microbiome.

### Fecal Sample Collection

Fecal samples were collected in a stool collection tube using the Stool Stabilizer (Stratec Molecular, Germany), separated, and stored at −80°C until microbial analysis.

### DNA Extraction, Internal Transcribed Spacer 1 rDNA Gene Amplification, and Sequencing

According to the manufacturer's instructions, the total microbial genomic DNA was extracted from biopsy samples using the PSP® Spin Stool DNA Kit (STRATEC Molecular, Germany). The fungal internal transcribed spacer 1 (ITS1) rDNA gene was amplified. In brief, the ITS1 region was amplified using the following primers: 5′-GGAAGTAAAAGTCGTAACAAGG-3′ (Forward, ITS5-1737F) and 5′-GCTGCGTTCTTCATCGATGC-3′ (Reverse, ITS2-2043R). The ITS rDNA gene was PCR-amplified in a 25-μl reaction volume containing 12.5 μl 2× KAPA HiFi HotStart ReadyMix (KAPA Biosystems, United States), 5 μl of each primer, 2.5 μl genomic DNA, and completed with ddH_2_O. The reaction was held at 95°C for 3 min, followed by 25 cycles at 95°C for 30 s, 55°C for 30 s, and 72°C for 30 s, with a final elongation step at 72°C for 5 min. Each PCR product was purified and amplified again to link with sample-specific barcodes.

### ITS1 rDNA Sequence Analysis

The leading software used for sequence analysis was Vsearch version 2.8.1 (Rognes et al., 2016) and Usearch version 10 (bit 64) (Edgar et al., 2011). The original data were merged by double-ended sequences using Vsearch, followed by data quality control, excision of primers, and barcode, and the 10,290 sequences were removed, leaving 6,230,337 sequences. Then, redundant sequences and sequences with <10 occurrences were removed using Vsearch. A total of 2,168,613 redundant sequences were removed, and 39,015 high-quality sequences were obtained.

Amplicon sequence variant (ASV) method was performed to filter chimeras (Edgar et al., 2011), and 4,191 high-quality amplicons were obtained. ASVs were aligned using the Vsearch and taxonomically classified using the reference sequence utax_reference_dataset_22.08.2016.fasta. All specimens were sampled into roughly the same amount (~40,000) of reads through Usearch V10, resulting in a total of 6,020,765 reads and 2,904 ASVs. Among them, 10 ASVs appeared in all samples, 34 ASVs appeared in 90%, and 147 ASVs occurred in 50% of samples (ASV ID in Supplementary Table).

### Statistical Analysis and Data Visualization

R 3.4.1 software with *ggplot2* package was used for visualization. Adonis test was performed for analysis of beta-diversity. The categorical variables were described by the number of cases, using the *chi*-square test or Fisher's exact test. The Shapiro–Wilk test tested the continuous variables, and the constant variables conformed to the normal distribution were described by the mean ± standard deviation (mean ± SD). The independent sample *t*-test/Mann–Whitney *U* non-parametric test compared the two groups. One-way ANOVA/Kruskal–Wallis H non-parametric test was used to compare the three groups. Correlation analysis was performed using the Spearman's test; *P*-value was corrected with a false discovery rate (FDR), and only significant correlations were visualized with the *pheatmap* package. Additionally, *P*-value ≤ 0.05 was considered to be statistically significant.

## Results

### Demographic and Clinical Data in Patients With PC

A 104 subjects were enrolled in this study, including 42 non-PC controls (CON) and 1:1 matched patients with PC (Table 1). Gender and age were reported to influence microbial profiles (Markle et al., 2013; Xu et al., 2022). To avoid gender- and age-based bias in fungal microbiota, we strictly enrolled gender- and age (±5)-matched subjects in two groups. Demographic and clinical data were collected for analysis, and no significant difference was found between patients with PC and CON subjects in comorbidities and laboratory parameters (Table 1).

**Table 1:** Demographic and clinical profiles of patients with PC and non-PC controls.

	**Non-PC controls (*n =* 52)**	**Post-cholecystectomy (*n =* 52)**	***p-*value**
Gender (M/F)	18/34	18/34	1.000
Age	59.71 (±11.95)	60.02 (±11.53)	0.735
BMI (kg/m^2^)	24.38 (±3.63)	25.71 (±3.47)	0.787
**Comorbidities**			
NAFLD	16 (30.8%)	22 (42.3%)	0.22
HBP	27 (51.9%)	27 (51.9%)	1.000
T2DM	15 (28.8%)	20 (38.5%)	0.299
HLP	10 (19.2%)	15 (28.8%)	0.326
CHD	3 (5.8%)	6 (11.5%)	0.244
**Laboratory tests**			
WBC (×10^9^/L)	6.10 (±1.59)	6.0469 (±1.47)	0.388
NE (%)	57.77 (±8.13)	56.00 (±8.87)	0.288
HB (g/L)	132.82 (±12.76)	137.83 (±14.022)	0.495
PLT (×10^9^/L)	232.22 (±53.60)	221.37 (±66.46)	0.392
ALT (U/L)	18.63 (±8.47)	21.37 (±9.34)	0.587
AST (U/L)	19.88 (±5.67)	21.87 (±7.27)	0.883
γ-GT (U/L)	29.02 (±22.53)	25.96 (±17.13)	0.285
ALP (U/L)	77.55 (±18.78)	85.32 (±32.18)	0.079
TBA (μmol/L)	3.2 (±0.58)	2.83 (±0.65)	0.846

### Fungal Diversity and Composition in PC Subjects

**Figure 1:**
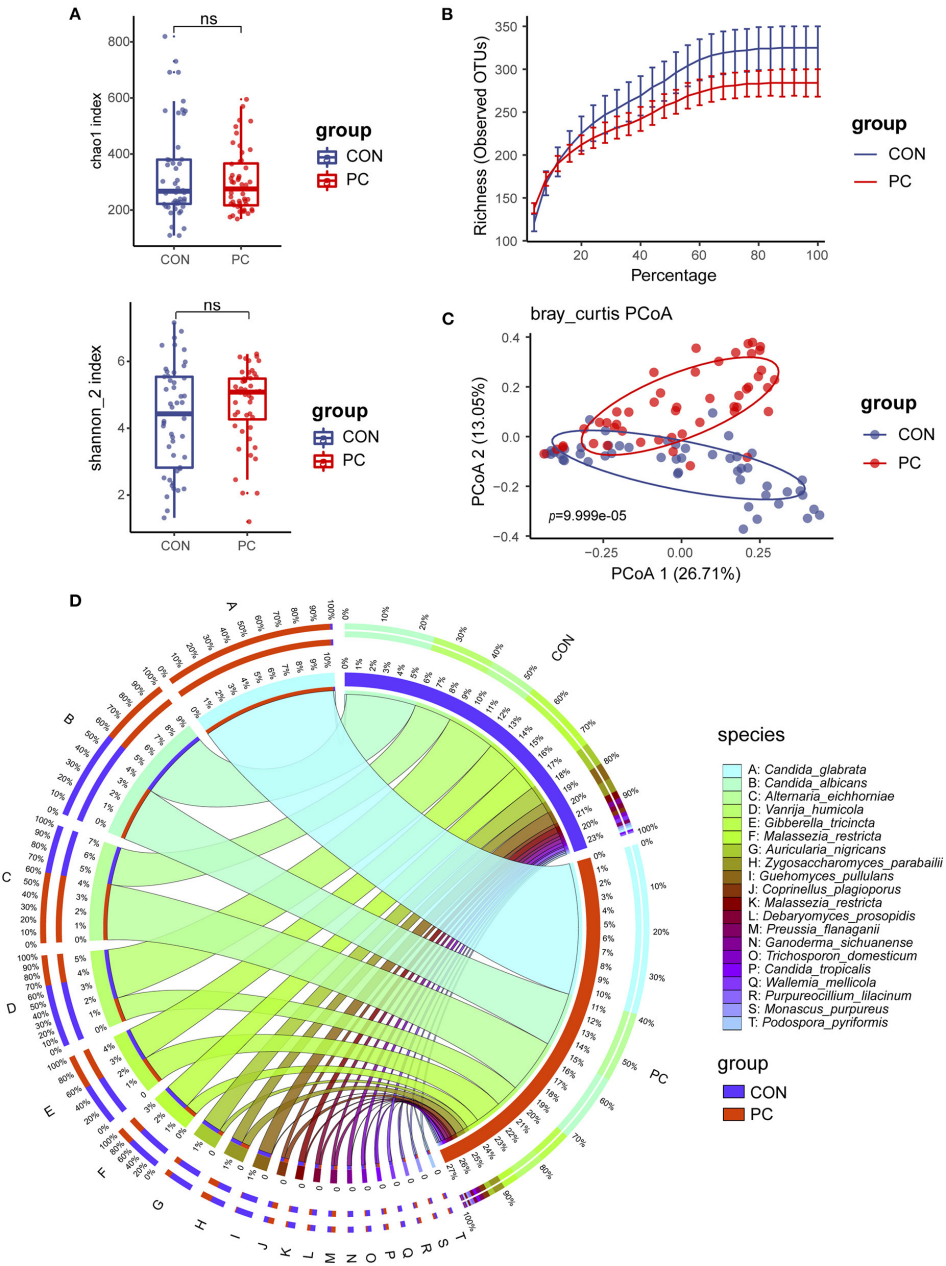
Fungal diversity and composition in PC and CON subjects. **(A)** Fungal alpha diversity based on Chao1 and Shannon index dealt with base 2 logarithm (log2, Shannon_2 index); ns, not significant. **(B)** Alpha rarefaction curve. **(C)** Beta diversity based on Bray–Curtis distance. **(D)** Fungal composition of top 20 species in CON and PC groups. Visualization was performed on the Circos website (http://circos.ca/). The color of the left outer and right inner bars represents groups (blue, CON; red, PC), and the color of the left inner bands and right outer bars represents fungal species. CON, non-PC control subjects; PC, postcholecystectomy. The left inner values represent the relative abundance of specific fungal contents in the total fungal community. The right inner values represent the sum value of these species in the CON or PC group.

We first compared the fungal alpha diversity in CON and PC subjects. We found that the Chao1 index was not significantly different when comparing both groups, nor Shannon, Richness, and Simpson's indexes (Figure 1A; Supplementary Figure 1A). These data indicated a similarity in fungal richness and evenness in these two groups of subjects. Although it was not significant, lower fungal ASVs were observed in the PC group (Figure 1B; Supplementary Figure 1B). Furthermore, beta diversity evaluated by Bray–Curtis distance showed a dramatic difference in fungal composition (Adonis *p* = 9.999e-05, Figure 1C); similar results were acquired from Jaccard and Weighted-UniFrac distance (Supplementary Figures 1C,D).

The fungal composition was further analyzed. Ascomycota and Basidiomycota were the two main phyla to constitute the fungal microbiota in all subjects at the phylum level (Supplementary Figure 1E). There was a higher abundance of Ascomycota and lower Basidiomycota in patients with PC (Supplementary Figure 1E). The species such as *Candida glabrata, Candida albicans, Alternaria eichhomiae, Vanrija humicola, Gibberella tricincta*, and *Malassezia restricta* were found to have higher mean abundance in all subjects (Supplementary Figure 1F). Of note, compared with the CON group, up to 95% of *C. glabrata* species were enriched in the PC group; additionally, a higher abundance of *Aspergillus eichhomiae* and a lower abundance of *V. humicola, M. restricta*, and *Auricularia nigricans* were in the PC group (Figure 1D).

These data suggested that the fungal alpha diversity was not significantly different, but the fungal composition was dramatically different in CON and PC subjects.

### Enriched and Depleted Fungal ASVs in Patients With PC

**Figure 2:**
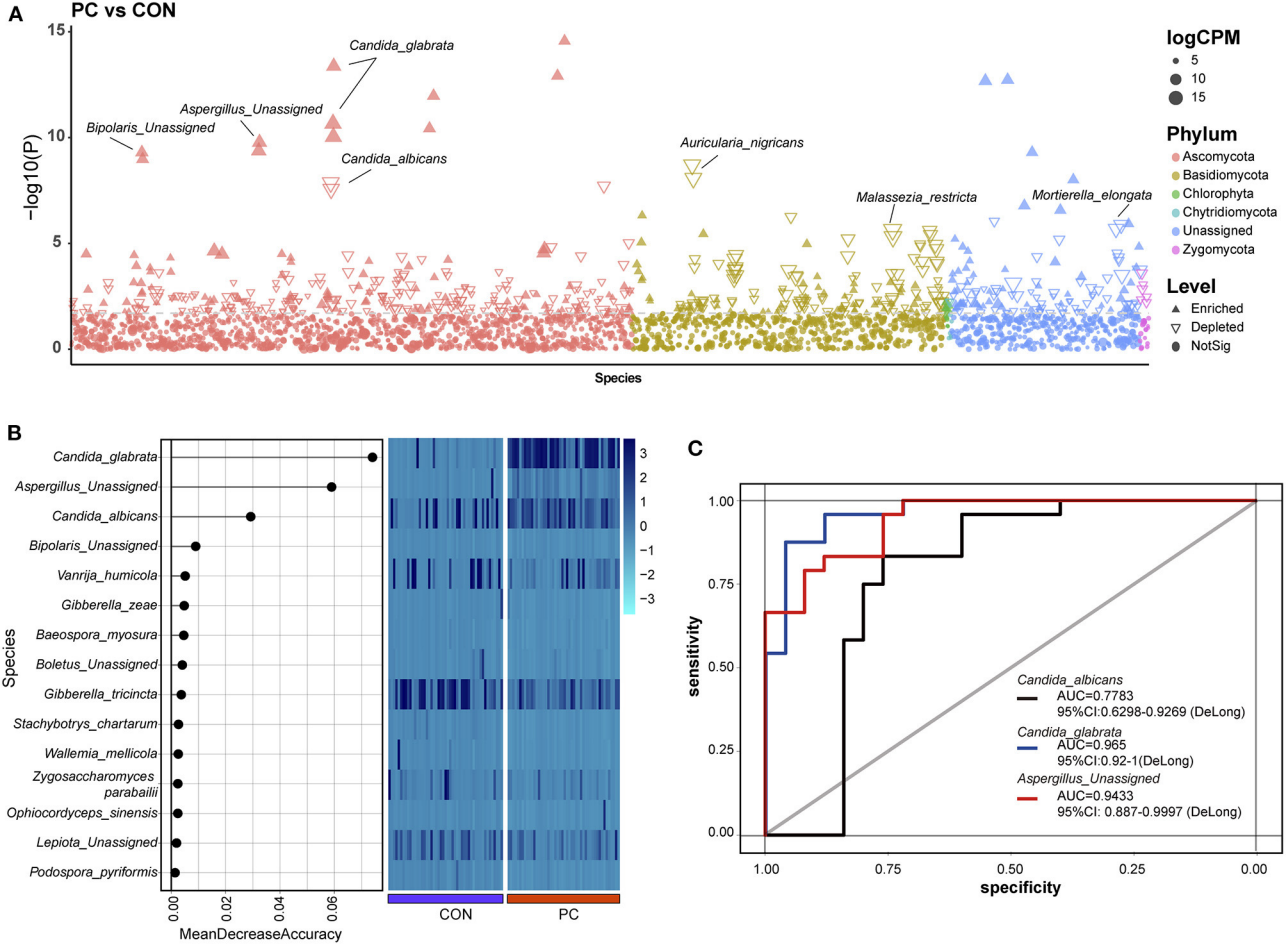
Enriched fungal ASVs and fungal biomarkers in patients with PC. **(A)** Enriched fungal ASVs in the PC group. EdgeR package was used for comparative analysis. The difference between the two groups is shown as a Manhattan diagram. Point shape indicates ASV enriched, depleted, or not significant in the former group compared with the latter one. Point color indicates fungal phylum. Point size indicates the abundance of ASV. CPM, count per million. **(B)** Machine learning based on random forest analysis to identify the fungal biomarkers. Fungal profiles from half of the subjects (*N*_CON_ = 26, *N*_PC_ = 26, randomly) were utilized for the model of machine learning. **(C)** Discrimination of patients with PC from CON subjects based on the fungal biomarkers. The receiving operational curve (ROC) analysis was performed on the rest of the subjects (*N*_CON_ = 26, *N*_PC_ = 26). Area under curve, AUC. The line colors represent the fungal biomarkers from the results of machine learning. Blue, *Candida sglabrata*; red, *Aspergillus Unassigned*; black, *Candida albicans*; CON, non-PC control subjects; PC, postcholecystectomy; ASVs, amplicon sequence variants.

To investigate the enriched or depleted fungal ASVs with a significant difference in patients with PC, we introduced *EdgeR* package to evaluate the ASV counts in the two groups. We found that *C. glabrata* (*P*_min_ = 4.26e-14, *Q*_min_ = 6.18e-11), *Aspergillus unassigned* (*P*_min_ = 1.67e-10, *Q*_min_ = 4.84e-08), and *Bipolaris unassigned* (*P*_min_ = 5.02e-10, *Q*_min_ = 1.12e-07) were significantly enriched in PC subjects. Additionally, *Aspergillus nigricans* (*P*_min_ = 1.95e-09, *Q*_min_ = 3.77e-07), *C. albicans* (*P*_min_ = 1.35e-08, *Q*_min_ = 2.17e-06), *V. humicola* (*P*_min_ = 2.27e-06, *Q*_min_ = 2.27e-04), and *M. restricta* (*P*_min_ = 2.47e-06, *Q*_min_ = 2.39e-04) were depleted in PC groups (Figure 2A; Supplementary Table 1).

Notably, 332 ASVs altered significantly in abundance (*P* < 0.01 and *Q* < 0.05), which indicated that the fungal microbiome underwent a dramatic alteration in patients with PC.

### Identification of Fungal Biomarkers in PC Subjects

Machine learning was used to identify microbial biomarkers (Tang et al., 2018; Ren et al., 2019). To explore the potential ability of fungal microbiota to discriminate patients with PC from CON subjects, we introduced a random forest classification model based on the profile of the fungal microbiome. Fungal profiles of half of the subjects in the two groups were picked out randomly for training, and then the learning results were confirmed in the rest half of the data. Three fungal species, including *C. glabrata, A. Unassigned*, and *C. albicans*, were identified as acceptable biomarkers (Figure 2B). We then assessed the performance of the identified fungal biomarkers using the receiver operating characteristic (ROC) curve. Data showed that the area under the curve (AUC) value of *C. glabrata* was 0.965 in patients with PC (95% CI: 0.92–1, Figure 2C). In addition, the AUC value of *A. Unassigned* and *C. albicans* achieved 0.9433 (95% CI: 0.887–0.9997) and 0.7783 (95% CI: 0.6298–0.9269), respectively (Figure 2C).

To validate this part of the result, the same dataset was used to analyze indicator species using the *Indicspecies* package (De Caceres and Legendre, 2009). Data also showed that *C. glabrata*, which was enriched in the PC group, was a specific fungal microbiota to indicator species (indicate value, IndVal = 0.993, *P* = 0.001, Supplementary Figure 2A). Additionally, the IndVal also presented *Guehomyces pullulans* as a potential indicator species (IndVal = 0.859, *P* = 0.001, Supplementary Figure 2A), but this species was not validated in machine learning. Taken together, the mentioned two methods confirmed that the *C. glabrata*-based classifier was able to distinguish patients with PC from control subjects, which highlighted the importance of the fungal species in the pathogenic process of patients with cholecystectomy.

### Environmental and Clinical Factors Associated With the Fungal Microbiome in Patients With PC

**Figure 3:**
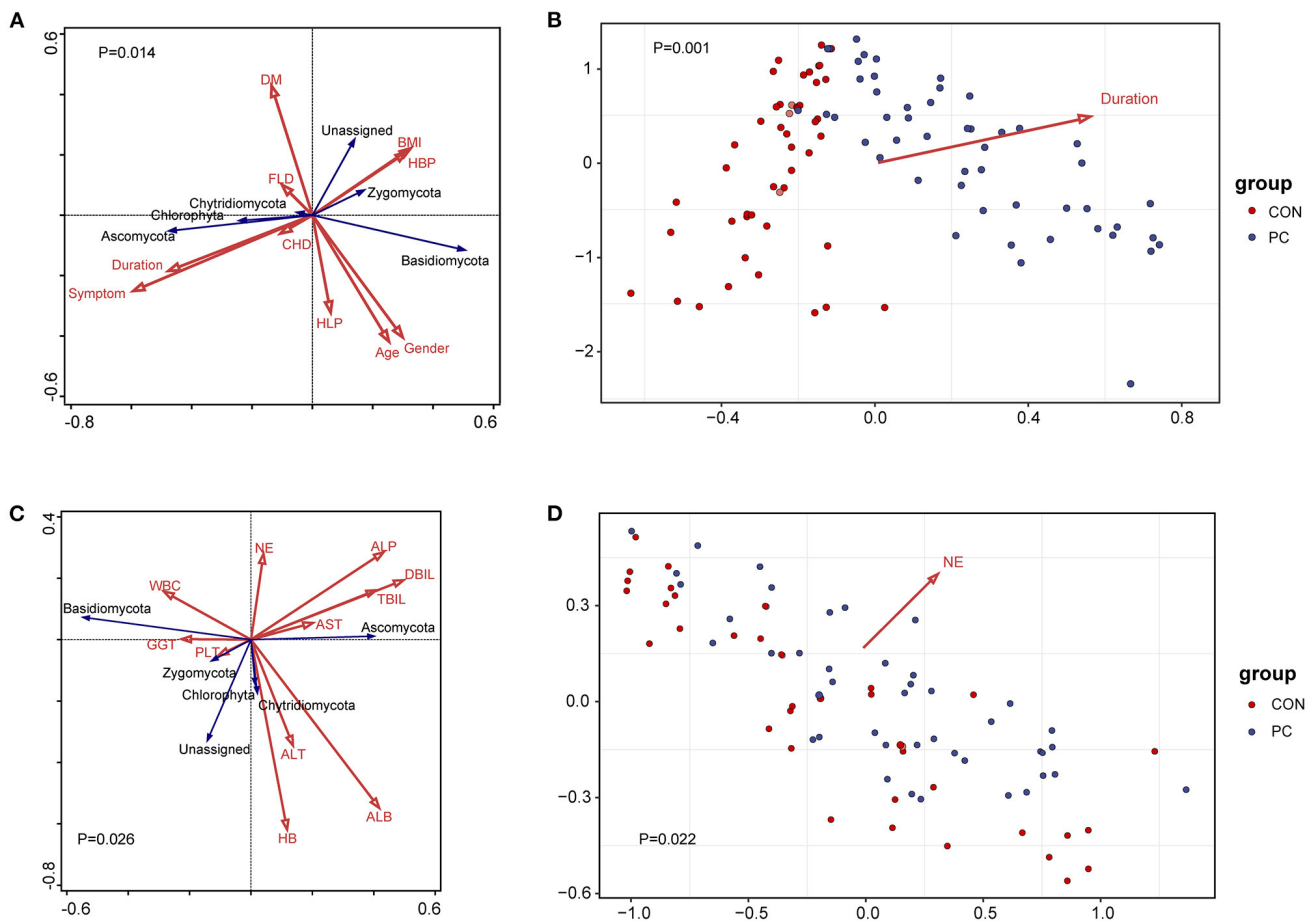
Environmental factors and clinical indexes associated with fungal microbiota. **(A)** Redundancy analysis (RDA) of environmental factors correlated with the fungal profile. The red arrow represents environmental factors and the blue represents fungal phyla. Results of the permutation test on all axes showed that pseudo-F equaled 2.2 with a *P*-value of 0.014. DM, diabetes mellitus; BMI, body mass index; HBP, high blood pressure; FLD, fatty liver disease; CHD, coronary heart disease; HLP, hyperlipemia; symptom, PC patients with gastrointestinal symptoms, including acid reflux, indigestion, bellyache, and diarrhea. Duration, years after patients with PC undergoing cholecystectomy. **(B)** Environmental factors fitting analysis. Only factors with significant correlation with sample clustering were noted as colored arrows in the panel. **(C)** RDA of clinical indexes correlated with the fungal profile. The red arrow represents clinical indexes and the blue represents fungal phyla. Results of the permutation test on all axes showed that pseudo-F equaled 1.8 with a *P*-value of 0.026. WBC, white blood cell; NE, neutrophilic granulocyte; ALP, alkaline phosphatase; AST, aspartate aminotransferase; ALT, alanine aminotransferase; HB, hemoglobin; PLT, platelet count; GGT, glutamyl transpeptidase; ALB, albumin; DBIL, direct bilirubin; TBIL, total bilirubin. **(D)** Clinical indexes fitting analysis. Only factors with significant correlation with sample clustering were noted as colored arrows in the panel.

A previous study showed that environmental factors affect the profiles of gastrointestinal microbiota (Smith et al., 2012; Song and Chan, 2019). To investigate which environmental factors are associated with the fungal microbiome in patients with PC, we recorded 10 factors, such as body mass index (BMI), age, gender, and duration after cholecystectomy, for analysis. To build the analysis model, we took fungal phyla into consideration in the redundancy analysis (RDA). We found that the model containing all factors was significantly associated with fungal microbiota (Pseudo-F = 2.2, *P* = 0.014, Figure 3A). Moreover, we further identified the most efficient environmental factor to drive fungal composition. Data showed that the duration after cholecystectomy was the most considerable factor to shape fungal microbiota (Pseudo-F = 4.6, *P* = 0.001, Figure 3B).

Additionally, we analyzed the correlation between clinical factors and fungal microbiome to primarily investigate the fungal influence on clinical parameters. As a result, 11 clinical indexes were collected in the RDA model, such as aspartate aminotransferase (AST), alanine aminotransferase (ALT), alkaline phosphatase (ALP), glutamyl transpeptidase (GGT), white blood cell count (WBC), and percentage of neutrophilic granulocyte (NE). Results showed that the fungal composition was significantly correlated with clinical indexes (Pseudo-F = 1.8, *P* = 0.026, Figure 3C). Further analysis showed that NE was the clinical factor closely associated with fungal microbiota (Pseudo-F = 1.75, *P* = 0.022, Figure 3D). We further analyzed the Spearman's correlation between specific fungal species and clinical parameters. Data showed that those indexes reflecting hepatic function, such as ALB, ALP, GGT, and AST, significantly correlated with various fungal species. Notably, the NE value was closely correlated with *C. glabrata*, enriched in PC subjects (Supplementary Figure 2B). These data suggested that fungal dysbiosis in patients with PC influenced host hepatic and immunological function, especially in the percentage of neutrophilic granulocyte.

### Fungal Composition in PC Patients With Precancerous Lesions or Colorectal Cancer

**Figure 4:**
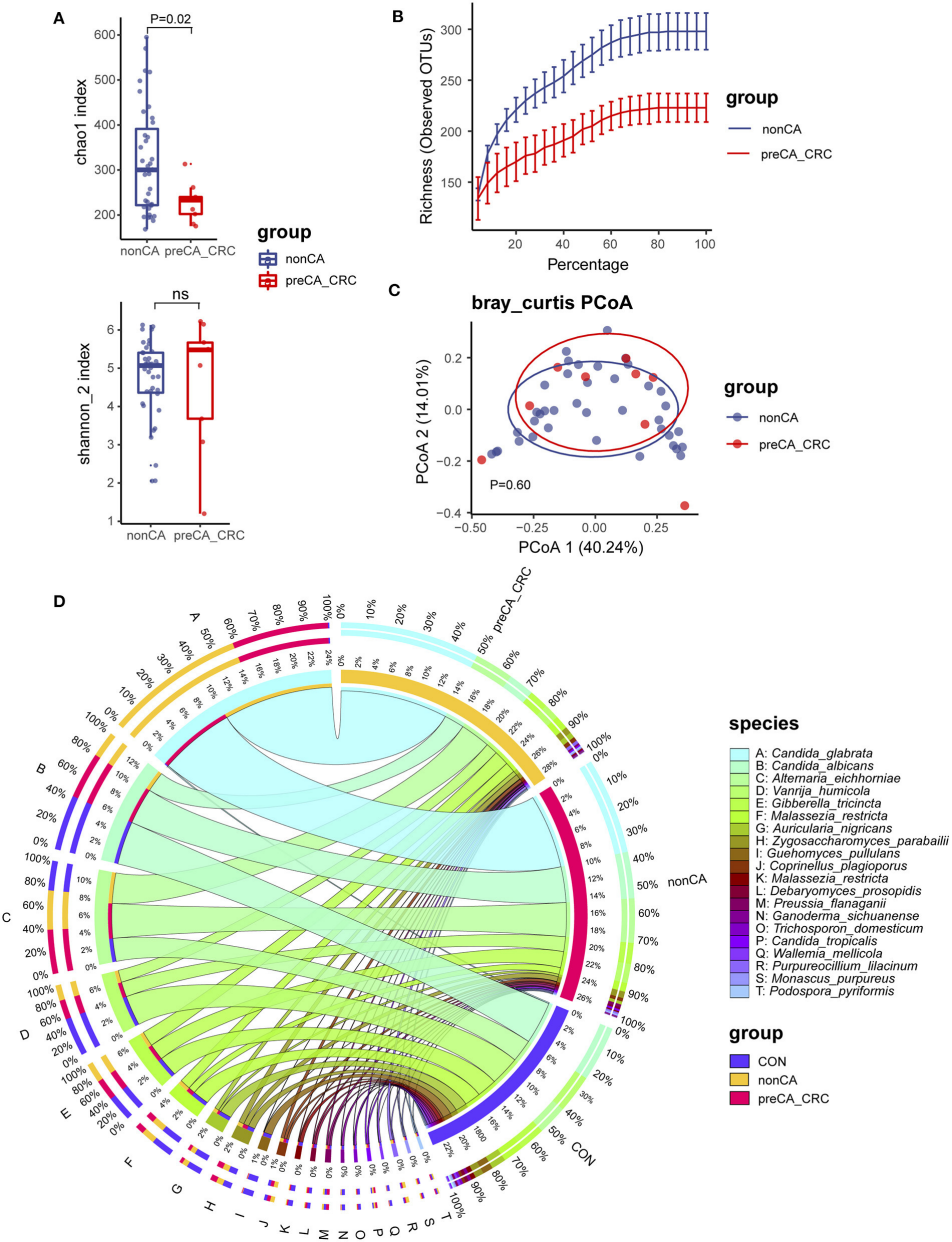
Fungal diversity and composition in PC patients with or without precancerous lesions and colorectal cancer. **(A)** Alpha diversity in patients with PC, displayed as chao1 index and Shannon index dealt with base 2 logarithm (log2, Shannon_2 index); ns, not significant. **(B)** Alpha rarefaction curve of two groups; **(C)** beta diversity based on Bray–Curtis distance. **(D)** Fungal composition of top 20 species in CON, non-CA (*N* = 43) and preCA_CRC (*N* = 9) groups. Visualization was performed on the Circos website (http://circos.ca/). The left inner values represent the relative abundance of specific fungal contents in the total fungal community. The right inner values represent the sum value of these species in the CON or PC group. CON, non-PC control subjects; non-CA, PC patients without precancerous lesions or colorectal cancer; preCA_CRC, PC patients with precancerous lesions or colorectal cancer.

Increasing evidence showed a high risk of patients with PC suffering colorectal cancer in long-term outcomes (Shao and Yang, 2005). To investigate the relationship between fungal microbiota and carcinogenesis in patients with PC, we divided patients with PC into non-CA and preCA_CRC subgroups based on histological evidence after colonoscopic sampling (Tables 2, 3). Fungal microbiota was compared between these subgroups. Fungal alpha diversity showed that there was a lower Chao 1 index (*P* = 0.02) in the preCA_CRC group (Figure 4A). Although it was not significant, lower fungal richness and Simpson index were also found in the preCA_CRC group (Supplementary Figure 3A). Notably, there was a lower observed ASV in the preCA_CRC group, which was accordant to chao1 and richness indexes (Figure 4B; Supplementary Figure 3B). Fungal beta diversity based on Bray–Curtis distance showed no distinguished clustering of samples in non-CA and preCA_CRC groups (*P* = 0.60, Figure 4C) and analysis based on weighted UniFrac and Jaccard distance (*P* = 0.536 and *P* = 0.402, respectively, Supplementary Figure 3C). These data indicated that the fungal microbiota underwent a significant alteration in PC patients with precancerous lesions and colorectal cancer.

**Table 2:** A new scoring for mucosal pathology in patients with postcholecystectomy.

**Mucosal pathological results**	**Post-cholecystectomy (*n =* 52)**	**Score**
No cancer sign	43 (82.7%)	0
Low grade intraepithelial neoplasia	6 (11.5%)	1
High grade intraepithelial neoplasia	1 (2.0%)	2
Invasive cancer	2 (3.8%)	3

**Table 3:** Demographic and clinical profiles of patients with non-CA and patients with preCA_CRC.

	**Non-CA patients**	**PreCA_CRC patients**	
	**(*n =* 43)**	**(*n* = 9)**	***p-*value**
Gender (M/F)	14/29	4/5	0.767
Age	58.44 (±11.386)	67.56 (±9.501)	0.030
BMI (kg/m^2^)	25.22 (±3.25)	28.08 (±3.71)	0.023
CRC family history	2 (4.7%)	1 (11.1%)	0.442
Duration	9.13 (±7.55)	11.17 (±10.34)	0.493
**Comorbidities**			
NAFLD	22 (51.2%)	5 (55.6%)	1.000
HBP	21 (48.8%)	6 (66.7%)	0.544
T2DM	16 (37.2%)	4 (44.4%)	0.977
HLP	11 (25.6%)	4 (44.4%)	0.465
CHD	5 (11.6%)	1 (11.1%)	1.000
**Laboratory tests**			
WBC (×10^9^/L)	5.91 (±1.49)	6.71 (±1.23)	0.388
NE (%)	55.62 (±8.92)	57.79 (±8.89)	0.288
HB (g/L)	137.56 (±14.56)	139.11 (±11.75)	0.495
PLT (×10^9^/L)	223.84 (±68.76)	209.56 (±56.09)	0.392
ALT (U/L)	20.98 (±8.61)	23.22 (±12.72)	0.587
AST (U/L)	21.91 (±6.93)	21.67 (±9.22)	0.883
γ-GT (U/L)	25.63 (±17.93)	27.33 (±14.03)	0.285
ALP (U/L)	86.32 (±33.23)	81.11 (±28.69)	0.079

We further investigated fungal composition in these groups. Ascomycota and Basidiomycota have also constituted the central part of fungal microbiota at the phylum level (Supplementary Figure 3D). Additionally, *Alternaria eichhorniae, A. nigricans, C. albicans*, and *C. glabrata* tended to be the most enriched fungal species in all patients (Supplementary Figure 3D). The top 20 species in all samples were picked for a percentage analysis (Figure 4D). Data showed that *C. glabrata, C. albicans, A. eichhorniae*, and *V. humicola* were the top four species in all subjects. Notably, few *C. glabrata* ASVs were presented in the CON group, while up to 99% of its ASVs were observed in patients with PC, including ~40% in non-CA and ~60% in the preCA_CRC subgroups (Figure 4D). These data suggested a gradually accumulation of *C. glabrata* species in long-term outcomes of patients after cholecystectomy and were also related to the carcinogenic process. Additionally, *C. albicans* and *V. humicola* were gradually depleted in non-CA and preCA_CRC subgroups.

To pick indicate species from the preCA_CRC group, we also performed the *Indicspecies* analysis and comparative analysis (Supplementary Figures 3E,F; Supplementary Table 2). We identified five fungal species, including *Wickerhamomyces anomalus* (IndicVal = 0.64, *P* = 0.021), *Curvularia lunata* (IndicVal = 0.64, *P* = 0.025), *Phaeophlebiopsis peniophoroides* (IndicVal = 0.47, *P* = 0.029), *Candida pseudoglaebosa* (IndicVal = 0.47, *P* = 0.034), and *Bjerkandera adusta* (IndicVal = 0.47, *P* = 0.036), which had the potential for discriminating patients with PC with precancerous lesions or colorectal cancer from patients without precancerous lesions or colorectal cancer (Supplementary Figure 3E). In addition, the comparative analysis also showed that *C. pseudoglaebosa* had a significant increase in patients of the preCA_CRC subgroup (*P*_min_ = 2.93e-5, *Q*_min_ = 0.028, Supplementary Figure 3F).

This part of the data showed a potential role of the *Candida* genus in the carcinogenic process of patients with PC.

## Discussion

In this study, we performed a match of the subjects in the PC and CON groups to avoid the gender- and age-based biases, which were reported to affect the microbial composition (Markle et al., 2013; Xu et al., 2022). We first compared the clinical parameters of patients with PC with CON subjects, and no significant differences were found. Based on these data and previous studies, it seemed reasonable for cholecystectomy to be an efficient, safe, and once-for-all therapeutic strategy for patients with biliary suffers (Li et al., 2014). However, increasing studies reported patients to happen to gastrointestinal comorbidities after cholecystectomy in a high risk, especially in colorectal cancer (Schernhammer et al., 2003; Shao and Yang, 2005; Simpson, 2019). Notably, bile acids and microbial profiles in patients with PC were dramatically undulated (Xu et al., 2001; Sauter et al., 2002; Wang T. et al., 2018), and their interaction was involved in colorectal inflammation and cancer (Li and Apte, 2015; Jia et al., 2018). We thus inferred that, as a result of cholecystectomy, bile acids without concentrating by the gallbladder pull down the intestinal lumen, which might be a driving factor for microbial dysbiosis. Subsequently, it is plausible that the resonance of the failure of bile acid biological rhythm and microbial dysbiosis tends to be the main factor in inducing colonic pathogenesis after cholecystectomy (Jia et al., 2018; Wang T. et al., 2018). Nowadays, many previous reports shed light on the fungal role in gastrointestinal pathogenesis, such as colitis and even carcinogenesis (Conche and Greten, 2018; Malik et al., 2018; Coker et al., 2019; Jun et al., 2020). Although the previous study showed the specific characterization of bacterial microbiota in patients with PC, few studies focused on the features of fungal microbiota. Thus, we focused on this point and investigated the fungal feature in patients with PC.

Depending on the alpha and beta diversity analysis, we found a dramatically different fungal profile in patients with PC (lower observed ASVs and specific composition), which was differentiated from CON subjects. Previous studies showed that the decrease in microbial diversity was related to various diseases' pathogenesis (Sokol et al., 2017; Coker et al., 2019). In all subjects of our research, Ascomycota and Basidiomycota were the dominant fungal phyla, while the abundant decrease of Basidiomycota in patients with PC also indicated a loss of fungal diversity, which was in accordance with previous studies.

The random forest-based analysis displayed that *C. glabrata, A. Unassigned*, and *C. albicans* were the species to discriminate patients with PC from CON subjects. Coker et al. reported that these kinds of *Aspergillus* sp. (such as *Aspergillus flavus, Aspergillus sydowii, Aspergillus ochraceoroseus*, and *Aspergillus rambellii*) were enriched in patients with CRC (Coker et al., 2019). The highly toxic carcinogen aflatoxin was reported to be produced by *Aspergillus* sp. (especially in *A. flavus*) (Arrieta et al., 2018). Consistent with previous research, our data also showed the possibility of *Aspergillus* in patients with PC to be involved in postoperative colorectal carcinogenesis. Additionally, *C. albicans* was one of the fungal pathogens to induce cancer development (Ramirez-Garcia et al., 2016; Muthular et al., 2019). Also, other *Candida* genera were reported to affect the process of carcinogenesis, such as *Candida tropicalis* and *C. glabrata* (Farmakiotis et al., 2014; Inacio et al., 2019). In this study, the abundance of *C. glabrata*, rather than *C. albicans*, was remarkably accumulated in patients with PC, and *C. pseudoglaebosa* tended to be an indicator species to discriminate preCA_CRC subgroup from patients with PC without precancerous lesions or colorectal cancer, which indicated a potential role of *Candida* genus in pathogenic progress of gastrointestinal postoperative carcinogenesis of patients with cholecystectomy. Interestingly, Wang et al. have illustrated that fungal burden, particularly of *Candida tropicals* but not *C. albicans*, is higher in human patients with CRC than in unaffected individuals (Conche and Greten, 2018; Wang T. et al., 2018), consistent with what we have found in our study. Additionally, non-*albicans Candida* (NAC), such as *C. tropicalis*, were considered less virulent due to a requirement for *Candida* biofilm formation in the initiation of infection and identified regulators of fungal morphogenesis (Efg1) and biofilm formation (Bcr1) (Yano et al., 2016); while *C. glabrata* did not undergo morphogenesis (Yano et al., 2019). As compensation, the Cst6p transcription factor was identified as a negative regulator of the EPA6 gene that encoded an adhesin central to *C. glabrata* biofilm formation (Riera et al., 2012). Furthermore, an effective metabolic adaptation strategy, modulating the glyoxylate cycle and alternative carbon metabolism, made it helpful for the survival and pathogenesis of *C. glabrata* (Chew et al., 2019). Notably, epidemiological data showed *C. glabrata* as an essential cause of candidemia in immunocompromised patients with cancer (Bodey et al., 2002; Hachem et al., 2008); while the role of *C. glabrata* in carcinogenesis, especially in the pathogenic process after cholecystectomy, remains unknown. Fungus-triggered immune response tended to be an underlying mechanism for host carcinogenesis. There was an essential role of CARD9-mediated antifungal immune response in maintaining intestinal stability and inducing intestinal carcinogenesis; data showed that CARD9 was involved in controlling fungal replication in macrophages (Bergmann et al., 2017; Conche and Greten, 2018; Drummond et al., 2018; Malik et al., 2018). Additionally, Wang W. et al. (2018) reported that NAC, such as *C. tropicals*, induced differentiation of immunosuppressive myeloid-derived suppressor cells (MDSCs), which suppresses T cell activation and mono-colonization of germ-free mice with *C. tropicalis* enhances tumor load. However, regarding the demographic features for non-CA and patients with preCA_CRC, we think it is essential for a further large-scale study to confirm whether these processes are involved in the *C. glabrata*-induced carcinogenesis.

Previous data showed that secondary bile acids inhibit *C. albicans* growth and morphogenesis (Guinan et al., 2018). Furthermore, the lipid components of bile acids increased fungal survival (including *C. albicans* and *Aspergillus terreus*) by enhancing the protective effect of conjugated bile salts against antifungal drugs (Hsieh and Brock, 2017). Our data also showed that the duration of cholecystectomy was the main factor affecting fungal microbiota. Taken together, we inferred that the alteration of bile acid profile after cholecystectomy tended to be a trigger for *Candida* and *Aspergillus* enrichment in the PC patient lumen.

Of note, the fungal community consists of a small part of the gut microbiome; however, the microbial interaction, such as bacteria-fungi interaction, was essential for microbial stabilization (Richard and Sokol, 2019; Jun et al., 2020). Our previous study also showed a dramatic alteration in the bacterial community in patients with PC (Ren et al., 2020). Like the human gut, Bacteroidetes can utilize fungal mannan through a selfish mechanism (Cuskin et al., 2015); we inferred that the fungal role also cross-talks with bacteria contents in triggering comorbidities in patients with PC.

In this study, we found fungal dysbiosis in patients with cholecystectomy and the postoperative duration as the potent factor influencing fungal composition. *Candida* (such as *C. glabrata* and *C. albicans*) and *Aspergillus* were biomarkers to discriminate patients with cholecystectomy from controls. The accumulation of *C. glabrata* might be connected with carcinogenesis after cholecystectomy.

## Data Availability Statement

The ITS1 sequences generated in this study are available through the NCBI Sequence Read Archive (Accession Number PRJNA541487).

## Ethics Statement

The studies involving human participants were reviewed and approved by Institutional Medical Ethics Review Board of Peking University People's Hospital. The patients/participants provided their written informed consent to participate in this study.

## Author Contributions

YulL and JX designed this study. XR, YuZ, YiZ, GC, QH, YunL, and QL performed sample and clinical information collection. JX, XR, and YunL conducted data analysis, visualization, and performed manuscript writing. YulL, JX, and XR revised this manuscript.

## Funding

This study was supported by the Beijing Municipal Natural Science Foundation (No. 7214267), the National Natural Science Foundation of China (Nos. 82070539, 81873549, and 82000496), and the Peking University People's Hospital Scientific Research Development Funds (Nos. RDY2020-21, RS2021-09, and RDL2021-11).

## Conflict of Interest

The authors declare that the research was conducted in the absence of any commercial or financial relationships that could be construed as a potential conflict of interest.

## Publisher's Note

All claims expressed in this article are solely those of the authors and do not necessarily represent those of their affiliated organizations, or those of the publisher, the editors and the reviewers. Any product that may be evaluated in this article, or claim that may be made by its manufacturer, is not guaranteed or endorsed by the publisher.

## Supplementary Material

Supplementary Figure 1 Fungal diversity and composition in PC and CON subjects. **(A)** Fungal alpha diversity based on Richness and Simpson's index; ns, not significant. **(B)** Alpha rarefaction curve for each sample. **(C)** Beta diversity based on Jaccard distance. **(D)** Beta diversity based on UniFrac distance; **(E)** Fungal composition in CON and PC groups at the phylum level. **(F)** The top 14 fungal species in CON and PC groups. CON, non-PC control subjects; PC, postcholecystectomy.

Supplementary Figure 2 Indicator fungal species and the correlation analysis between fungal species and clinical data in all subjects. **(A)** Indicator species in CON and PC groups. *Indicspecies* package was used for indicator fungal species analysis. Point shape represents ASV enriched (high) or depleted (low) in the group. Point color represents the groups of all subjects. Point size indicates the abundance of fungal ASVs. IndVal, indicator value. The value of IndVal is in the range of 0–1; the value is bigger, the more powerful is the indicator for differentiating the two groups. CON, non-PC control subjects; PC, postcholecystectomy; ASVs, amplicon sequence variants. **(B)** The correlation analysis between fungal species and clinical data in all subjects. *Pheatmap* package was used for the correlation analysis. The *P*-value of correlation analysis was corrected with the false discovery rate (FDR), and only significant correlations were labeled as stars; ^*^FDR ≤ 0.05; ^**^FDR ≤ 0.01; ^***^FDR ≤ 0.001. WBC, white blood cell; NE, neutrophilic granulocyte; ALP, alkaline phosphatase; AST, aspartate aminotransferase; ALT, alanine aminotransferase; HB, hemoglobin; PLT, platelet count; GGT, glutamyl transpeptidase; ALB, albumin; DBIL, direct bilirubin; TBIL, total bilirubin.

Supplementary Figure 3 Fungal diversity and composition in non-CA and preCA_CRC subgroups. **(A)** Fungal alpha diversity based on Richness and Simpson's index; ns, not significant. **(B)** Alpha rarefaction curve for each sample. **(C)** Beta diversity based on UniFrac distance and Jaccard distance; **(D)** fungal composition in non-CA and preCA_CRC subgroups at the phylum level (**D**, upper panel), and at the species a level (top 15, **D**, lower panel). **(E)** Indicator species in non-CA and preCA_CRC subgroups. *Indicspecies* package was used for indicator fungal species analysis. Point shape represents ASV enriched (high) or depleted (low) in the group. Point color represents the subgroups of patients with PC. Point size indicates the abundance of fungal ASVs. IndVal, indicator value. The value of IndVal is in the range of 0–1; the value is bigger, and the more powerful is the indicator for differentiating the two groups. The symbols after the Indic values represent statistical significance ^*^, *P* < 0.05; ., *P* < 0.1. **(F)** Enriched fungal ASVs in the preCA_CRC subgroup. EdgeR package was used for comparative analysis. The difference between the two groups was shown as a Manhattan diagram. Point shape indicates ASVs enriched or not significant in the former group compared with the latter one. Point color indicates fungal phylum. Point size indicates the abundance of ASV. CPM, count per million. Non-CA, postcholecystectomy patients without precancerous lesions or colorectal cancer; preCA_CRC, patients with postcholecystectomy complicated with precancerous lesions or colorectal cancer; ASVs, amplicon sequence variants.

Supplementary Table 1 Fungal ASVs with significant difference (PC vs. HC).

Supplementary Table 2 Fungal ASVs with significant difference (preCA_CRC vs. non-CA).

## Abbreviations

ALP: alkaline phosphatase
ALT: alanine aminotransferase
AST: aspartate aminotransferase
BMI: body mass index
BSH: bile salt hydrolase
CHD: coronary atherosclerotic heart disease
CRC: colorectal cancer
DCA: deoxycholic acid
ASVs: amplicon sequence variants
GGT: glutamyl transpeptidase
HB: hemoglobin
HBP: hypertension
HLP: hyperlipemia
LCA: lithocholic acid
NAFLD: non-alcoholic fatty liver disease
NE: neutrophilic granulocyte percentage
PCoA: principal coordinate analysis
PLT: blood platelet count
SP-D: surfactant protein D
SCFAs: short-chain fatty acids
STAMP: statistical analysis of taxonomic and functional profiles
T2DM: type 2 diabetes mellitus
TBA: total bile acid
WBC: white blood cell count.
