# Supplementary figures and images for: Alterations of Fungal Microbiota in Patients With Cholecystectomy
Source: Front Microbiol. 2022 May 11;13:831947. doi: 10.3389/fmicb.2022.831947 (PMC9132483; doi:10.3389/fmicb.2022.831947)

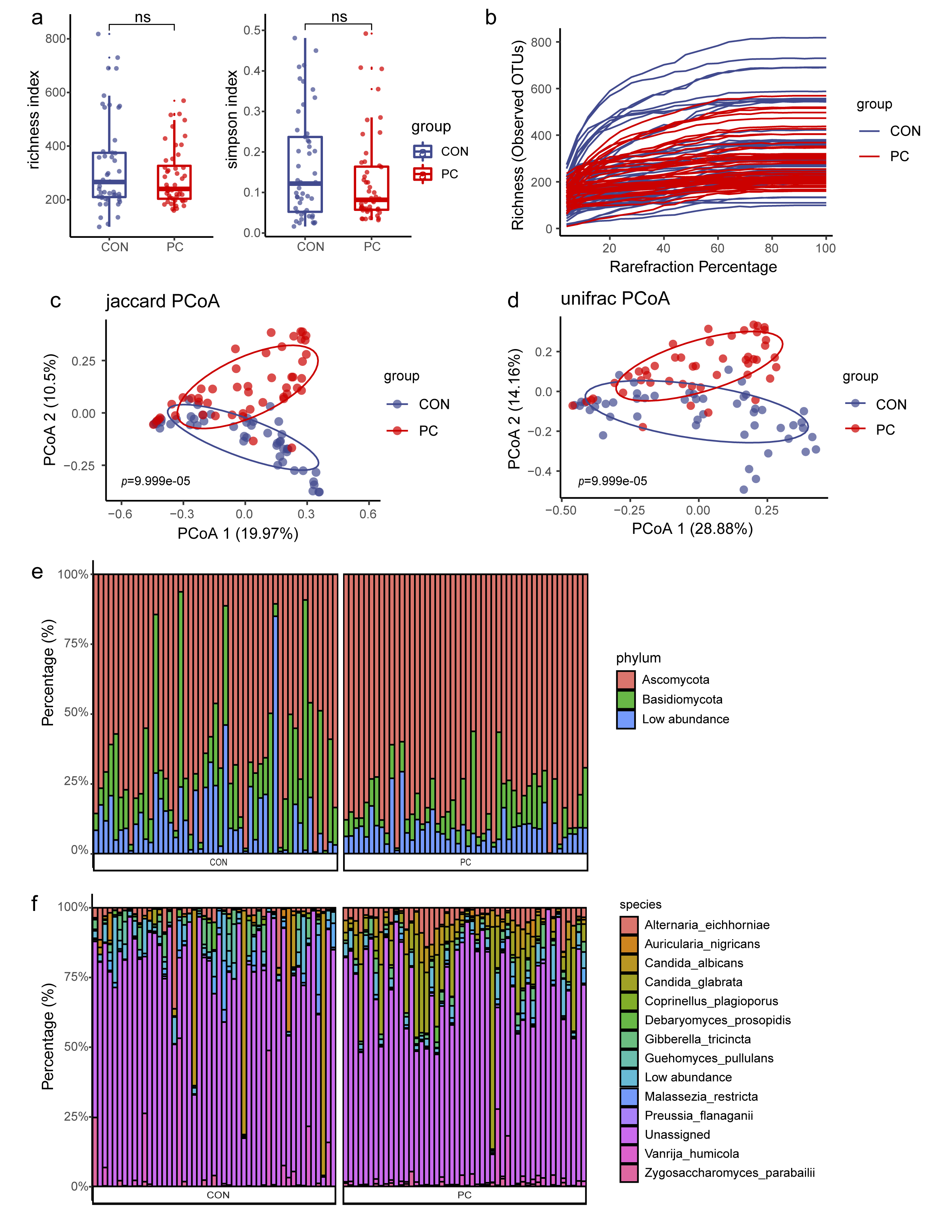

Supplement: Supplementary Figure 1 — Fungal diversity and composition in PC and CON subjects. (A) Fungal alpha diversity based on Richness and Simpson's index; ns, not significant. (B) Alpha rarefaction curve for each sample. (C) Beta diversity based on Jaccard distance. (D) Beta diversity based on UniFrac distance; (E) Fungal composition in CON and PC groups at the phylum level. (F) The top 14 fungal species in CON and PC groups. CON, non-PC control subjects; PC, postcholecystectomy. [file Image_1.TIF]

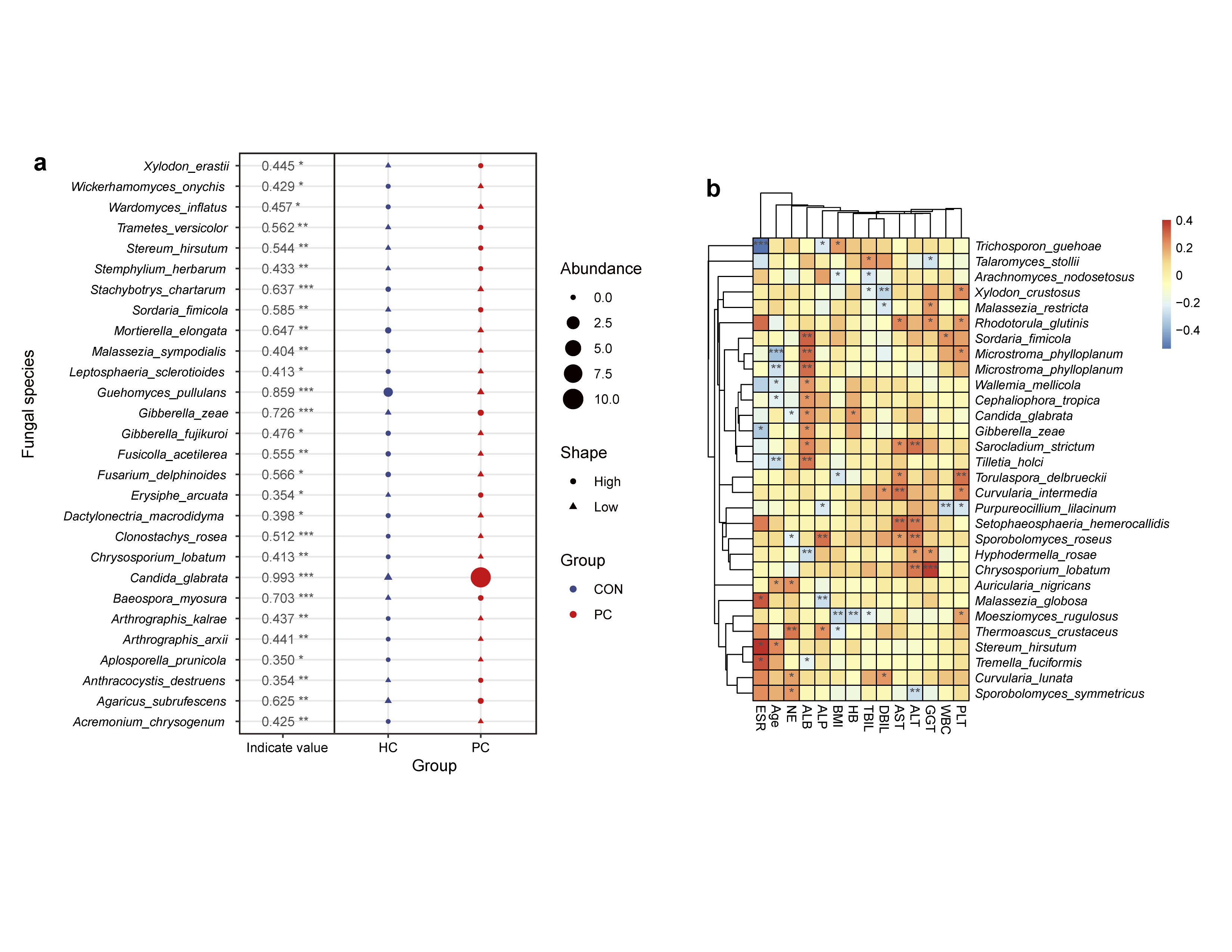

Supplement: Supplementary Figure 2 — Indicator fungal species and the correlation analysis between fungal species and clinical data in all subjects. (A) Indicator species in CON and PC groups. Indicspecies package was used for indicator fungal species analysis. Point shape represents ASV enriched (high) or depleted (low) in the group. Point color represents the groups of all subjects. Point size indicates the abundance of fungal ASVs. IndVal, indicator value. The value of IndVal is in the range of 0–1; the value is bigger, the more powerful is the indicator for differentiating the two groups. CON, non-PC control subjects; PC, postcholecystectomy; ASVs, amplicon sequence variants. (B) The correlation analysis between fungal species and clinical data in all subjects. Pheatmap package was used for the correlation analysis. The P-value of correlation analysis was corrected with the false discovery rate (FDR), and only significant correlations were labeled as stars; *FDR ≤ 0.05; **FDR ≤ 0.01; ***FDR ≤ 0.001. WBC, white blood cell; NE, neutrophilic granulocyte; ALP, alkaline phosphatase; AST, aspartate aminotransferase; ALT, alanine aminotransferase; HB, hemoglobin; PLT, platelet count; GGT, glutamyl transpeptidase; ALB, albumin; DBIL, direct bilirubin; TBIL, total bilirubin. [file Image_2.TIF]

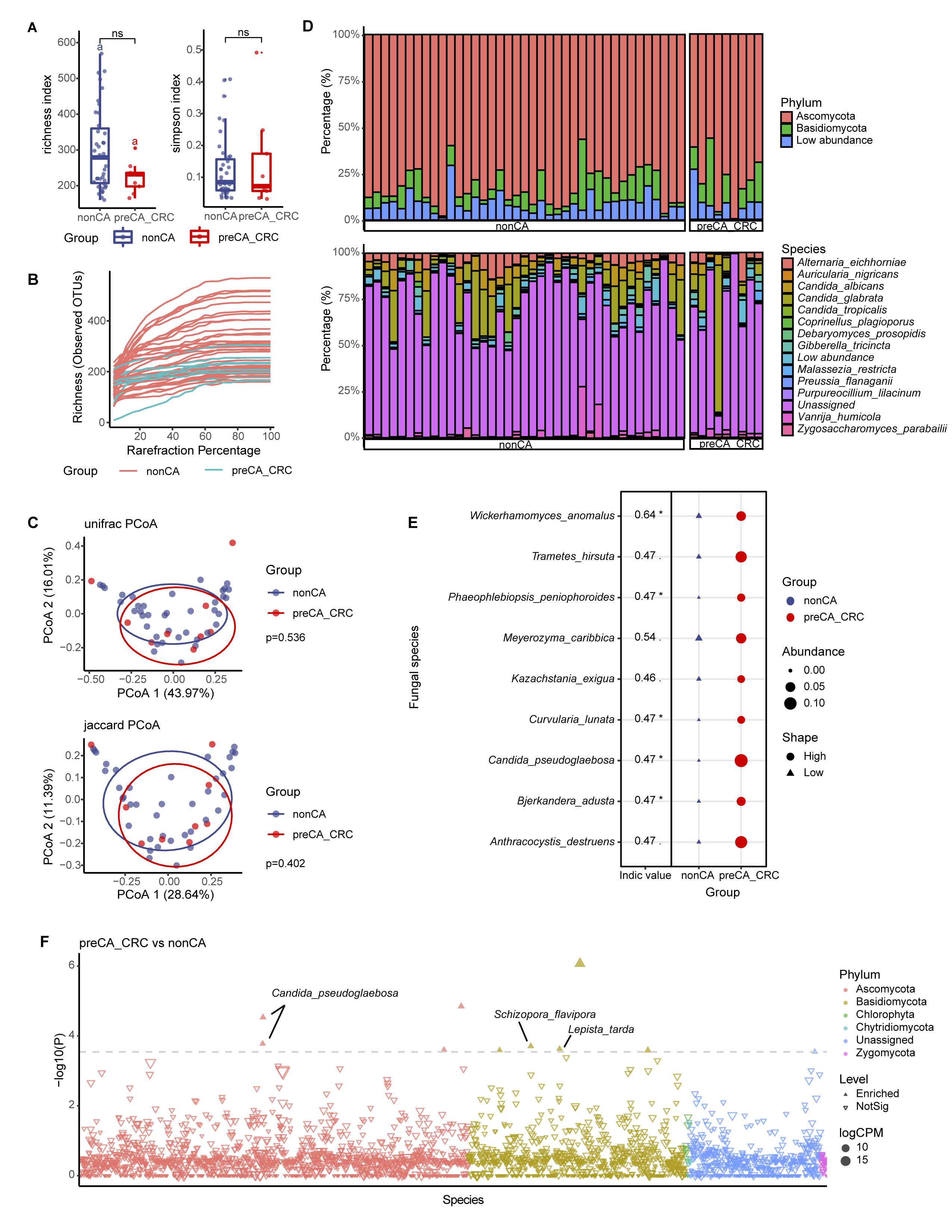

Supplement: Supplementary Figure 3 — Fungal diversity and composition in non-CA and preCA_CRC subgroups. (A) Fungal alpha diversity based on Richness and Simpson's index; ns, not significant. (B) Alpha rarefaction curve for each sample. (C) Beta diversity based on UniFrac distance and Jaccard distance; (D) fungal composition in non-CA and preCA_CRC subgroups at the phylum level (D, upper panel), and at the species a level (top 15, D, lower panel). (E) Indicator species in non-CA and preCA_CRC subgroups. Indicspecies package was used for indicator fungal species analysis. Point shape represents ASV enriched (high) or depleted (low) in the group. Point color represents the subgroups of patients with PC. Point size indicates the abundance of fungal ASVs. IndVal, indicator value. The value of IndVal is in the range of 0–1; the value is bigger, and the more powerful is the indicator for differentiating the two groups. The symbols after the Indic values represent statistical significance *, P < 0.05; ., P < 0.1. (F) Enriched fungal ASVs in the preCA_CRC subgroup. EdgeR package was used for comparative analysis. The difference between the two groups was shown as a Manhattan diagram. Point shape indicates ASVs enriched or not significant in the former group compared with the latter one. Point color indicates fungal phylum. Point size indicates the abundance of ASV. CPM, count per million. Non-CA, postcholecystectomy patients without precancerous lesions or colorectal cancer; preCA_CRC, patients with postcholecystectomy complicated with precancerous lesions or colorectal cancer; ASVs, amplicon sequence variants. [file Image_3.TIF]
